# A Review of Commercial and Non-Commercial Wearables Devices for Monitoring Motor Impairments Caused by Neurodegenerative Diseases

**DOI:** 10.3390/bios13010072

**Published:** 2022-12-31

**Authors:** Guillermo Prieto-Avalos, Laura Nely Sánchez-Morales, Giner Alor-Hernández, José Luis Sánchez-Cervantes

**Affiliations:** 1 Tecnológico Nacional de México/I.T. Orizaba, Av. Oriente 9 No. 852 Col. Emiliano Zapata, Orizaba 94320, Veracruz, Mexico; 2 CONACYT-Tecnológico Nacional de México/I.T. Orizaba, Av. Oriente 9 No. 852 Col. Emiliano Zapata, Orizaba 94320, Veracruz, Mexico

**Keywords:** neurodegenerative diseases, monitoring, sensors, wearables

## Abstract

Neurodegenerative diseases (NDDs) are among the 10 causes of death worldwide. The effects of NDDs, including irreversible motor impairments, have an impact not only on patients themselves but also on their families and social environments. One strategy to mitigate the pain of NDDs is to early identify and remotely monitor related motor impairments using wearable devices. Technological progress has contributed to reducing the hardware complexity of mobile devices while simultaneously improving their efficiency in terms of data collection and processing and energy consumption. However, perhaps the greatest challenges of current mobile devices are to successfully manage the security and privacy of patient medical data and maintain reasonable costs with respect to the traditional patient consultation scheme. In this work, we conclude: (1) Falls are most monitored for Parkinson’s disease, while tremors predominate in epilepsy and Alzheimer’s disease. These findings will provide guidance for wearable device manufacturers to strengthen areas of opportunity that need to be addressed, and (2) Of the total universe of commercial wearables devices that are available on the market, only a few have FDA approval, which means that there is a large number of devices that do not safeguard the integrity of the users who use them.

## 1. Introduction

Neurodegenerative diseases (NDDs) affect the central nervous system as they destroy the neuronal cells found there. Some of these diseases are epilepsy, Parkinson’s disease (PD), and Alzheimer’s disease (AD), among others. In turn, neuronal deterioration affects different organs and activities, such as balance, speech, breathing, and heart function. NDDs can also cause cognitive deficits, dementia, and behavioral disorders [1]. In 2019, the World Health Organization (WHO) reported that 55% of the 55.4 million deaths that occurred worldwide were identified in the ten leading causes of death [2,3]. Currently, seven of the ten leading causes of death are non-communicable diseases. This number constitutes a considerable increase if compared to 2000, when non-communicable diseases were four of the top 10 causes of death. Nowadays, NDDs such as AD are already among the 10 leading causes of death worldwide and have ranked third in both the Americas and Europe since 2019 [3,4].

Around 50 million people worldwide are affected by NDDs, of which more than half live in countries considered low- and middle-income. Approximately 10 million new cases are registered annually, and it is estimated that between 5% and 8% of the general population aged 60 or over will suffer from some NDDs [3], and by 2030 and 2050, respectively, 78 and 139 million patients are expected to have some NDDs [5]. There is no cure or reversal of the progressive evolution of NDDs; however, there is research on new treatments that can be offered to support and improve the lives of patients, as well as their caregivers and relatives [6].

The relationship between cognitive impairment and mobility has been widely studied and recognized [7]. The different NDDs share symptoms of progressive cognitive deterioration, which also impacts the motor functions of the patient, such as walking gait and balance; therefore, continuous monitoring of these motor functions helps to diagnose and assess the severity of neurological disorders. This has motivated technological development (Apps, wearable devices, among others) in order to provide relevant and precise metrics on the motor functions of patients with some NDDs [8].

A wearable device is any electronic device capable of processing and storing information, which has been incorporated into the clothing or accessories that a person uses on their body on a daily basis; for example, watches, bracelets, glasses, caps, headbands, shirts, and shoes, among others [9]. Among the sectors where it is common to use wearable devices are industrial, military, and health, and because its operation is based on acquiring and processing data to make a decision based on it, it is to be expected that they have an operating system built into the microcomputer found incorporated in the wearable device [10]. The Android operating system is recognized to be used in more than 70% of smartphones worldwide and is easy to use [11]. This open-source operating system based on the Linux kernel offers great flexibility due to its customization properties, making it a dominant mobile operating system. It is increasingly common to find Android smartphones, wearable devices and portable devices with healthcare applications installed [12]. As an example of the above, there are commercial wearable devices that work together with devices with the Android Operating System (for example, a cell phone) to generate a user database [13]. A wearable device must have the characteristic of being imperceptible to the user; that is to say, to interfere as little as possible in the activities carried out daily and at the same time to be efficient and precise in the information provided by the user [14]. Currently, the manufacture of wearable devices that have this characteristic is under development; as it is, the technology is based on tattoos, smart textiles, and biosensors [15]. Wearable devices have made it possible to monitor aspects of health outside of a clinical setting, favorably impacting clinical care and self-monitoring of symptoms and, therefore, the health of the patient in general. This has also benefited patients who live in remote regions or far from specialized clinics. Remote monitoring has been shown to be useful in recording behaviors that occur sporadically outside of a clinical setting or in patients exhibiting time-varying behaviors or presenting particular symptoms in the clinic compared to home [16].

### Common Motor Impairments Caused by NDDs

**Figure 1:**
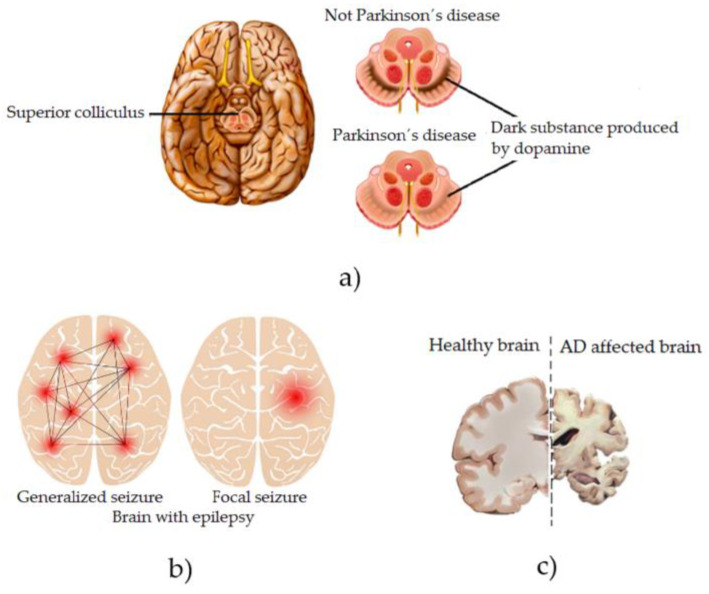
Graphic representation of the effects on the brain of some NDDs. (**a**) Parkinson´s disease, (**b**) Epilepsy and (**c**) Alzheimer disease.

NDDs gradually kill the patient’s motor neurons, thus affecting essential muscle activities such as talking, walking, breathing, and swallowing [17]. They cause gradual muscle weakness, uncontrollable tics, muscle or limb rigidity, and spasms on the knee and ankles, finally causing the patient to lose their ability to voluntarily control their movements [18]. In patients diagnosed with PD, specific areas of their brain deteriorate, and significant changes occur in their brain chemistry, for example, a lack of dopamine [19], which is graphically illustrated in Figure 1a. As the PD progresses, the symptoms that appear in the patient are uncontrollable tremors, slow movements in their movements (bradykinesia), voice and speech disorders, changes in posture and balance, and muscle rigidity, among others [20]. As the disease progresses, all of these symptoms often expand and intensify throughout the patient’s body, causing depression and dementia-like symptoms [21]. Patients diagnosed with Epilepsy (Neurological Disorder) are characterized by having recurrent and unpredictable seizures when the normal electrical signaling between neurons in their brain is disrupted [22], which is graphically illustrated in Figure 1b. Among the symptoms found in this disease are temporary confusion and, therefore, episodes of absences, rigidity in the muscles of the whole body producing uncontrollable spasmodic movements mainly in the arms and legs, and psychological affectations, such as fear or anxiety [23]. In the event that the disease is not controlled under medical surveillance and its progression is allowed to advance, the patient’s brain suffers neurological alterations over time, irreversibly affecting their motor and cognitive skills [24]. Patients diagnosed with AD have a brain disorder that slowly destroys memory and the ability to think and, over time, ceases to be independent when performing daily activities [25], which is graphically illustrated in Figure 1c. Among the symptoms of this disease are cognitive impairment, temporary and spatial disorientation, and difficulty in expressing oneself, among others [26]. The havoc in the patient’s body as the disease progresses is reflected in leaving him practically unable to perform any physical or cognitive activity considered essential in any person [27].

**Figure 2:**
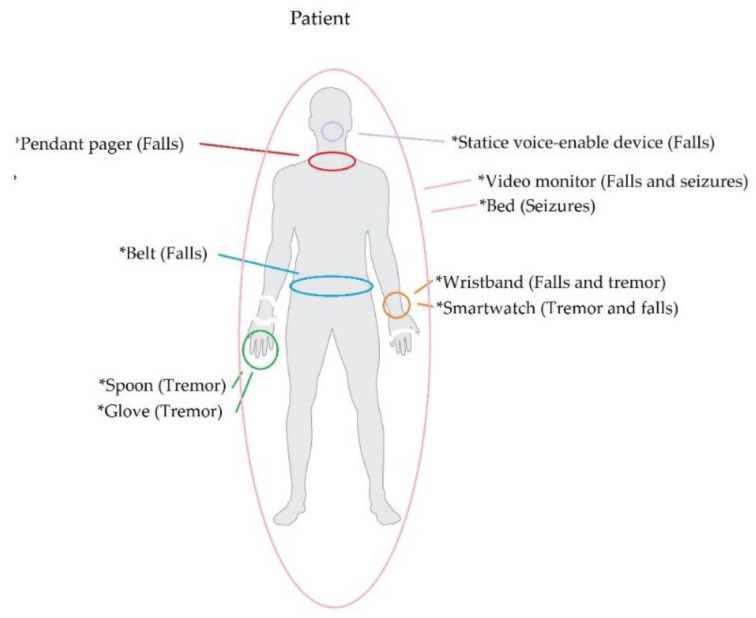
Types of wearable devices and the impairments that they are typically used to detect.

Examining NDDs through wearable technology involves monitoring related motor impairments, such as tremors, falls, and seizures. In the following paragraphs, we summarize the NDD-related motor impairments that are monitored via wearable devices. Then, in Figure 2, we illustrate the parts of the human body that are commonly associated with such devices.

*Tremors:* They appear when the person is at rest, regularly and rhythmically, disappearing when they change posture or make movements. At early stages, tremors commonly occur on one side of the upper body (often one hand). This motor symptom, similar to the rest, evolves over time: (1) it first manifests itself in the upper limbs (although it can occur in the lower limbs, lip, chin, or tongue) and on one side of the body in a specific area (commonly the hand). (2) It progresses through the extremity and usually passes to the lower area, foot, and leg, on the affected side. Finally, (3) it evolves on both sides of the body. The voice and head are rarely affected. The severity of the tremors also depends on the time of the day. Fatigue, anxiety, and concentration make them more intense, while during sleep hours, they disappear. This affectation affects not only the patient but also their family, work, and/or social environments [28]. Technological advancement has led to the development of motion sensors that are wireless and wearable. Accelerometer, gyroscopic, and electromyography (EMG) are just some of the sensing technologies used to record patient tremor data [29].

*Falls:* The most common symptoms of NDDs include rigidity, bradykinesia, and posture, which contribute to the patient’s risk of falling. Rigidity means little flexibility in the movement of the neck and trunk, which results in a loss of balance, thus increasing the likelihood of a patient falling. A person’s center of gravity is below the navel, and the legs form the base of support. Hence, problems with the center of gravity contribute to a fall [30]. Loss of balance might occur while doing a regular activity, such as standing up, bending over, walking, turning the head, avoiding obstacles, or talking. Falls can also occur due to impaired postural reflexes, irregular changes in posture, and episodes of sudden paralysis while walking. Other risk factors in falls are the patient’s vision and depth perception problems [31]. The technologies used to detect falls include motion sensors, sound sensors, and video cameras. They collect data on the patient’s movement activity. Then, using algorithms, they analyze and determine a patient’s activity level to accurately detect a fall. Usually, motion and sound sensors are installed in fixed locations; however, some researchers have proposed their use in the form of a wearable device to monitor patient movement patterns [32].

*Seizures* are uncontrolled and sudden electrical disturbance in the brain that manifest as changes in patient behavior, motor movements, and levels of consciousness. Having more than one seizure in a 24-h period with no apparent cause is considered epilepsy. There are several types of seizures, with different symptoms and levels of severity, which vary depending on the region of the brain in which they start and end [33]. The duration of a seizure is commonly 30 to 120 s. If the seizure lasts more than five minutes, it is considered a medical emergency and causes the person to fall over, shake, and be unaware of what is going on around them [34]. Accelerometry is widely used to detect seizures that are expressed in movements or alter normal movement patterns. Three-dimensional accelerometers, algorithms based on Hidden Markov Models (HMM), and Bayesian analysis are just some of the proposals to optimally detect seizures in patients [35].

The rest of this document is organized into the following sections: (1) objective and justification of this research; (2) explained the methods; (3) research questions, the search strategy, the selection process of primary studies and data extraction; (4) present the results of the review; (5) discussion of the results; and (6) conclusions.

## 2. Research Goal and Need for Literature Review

The literature reports a substantial number of scientific contributions in the management, follow-up, and detection of NDDs, including monitoring technologies, patient behavior analysis, and technology development. Researchers such as Kristoffersson et al. [36], Mughal et al. [37], Sica et al. [38], Celik et al. [39], Cicirelli et al. [8], Albán-Cadena et al. [40], Storm et al. [41], Zampogna et al. [42], Rehman et al. [43], Godoi et al. [44], Chirra et al. [45], Brognara et al. [46], Rovini et al. [47], Díaz et al. [48], Porciuncula et al. [49], Silva de Lima et al. [50], Vienne et al. [51], Wang et al. [52], Giggins et al. [53], Hubble et al. [54], Oung et al. [55], and Steins et al. [56] based on wearable sensors and machine learning (ML) models, proposed optimizing the monitoring and analysis of physical activity in patients with PD, to assess the quality of gait and balance maintained by the patient in question. In all of these cases, relevant data have been obtained to establish rehabilitation routines, the prognosis of falls, and monitoring disease progression.

In their work, Stavropoulos et al. [57] and Pasluosta et al. [58] evaluated the opportunities and challenges of wearable sensors with access to the Internet of Things (IoT) and applied them to the care of patients with Parkinson’s disease. Researchers Tăuţan et al. [59], Ghannam et al. [60], Buckley et al. [61], and Belić et al. [62] implemented Artificial Intelligence (AI) and evaluated the opportunities and challenges through ML techniques and Big Data in wearable devices for the care of patients with PD. In turn, Sivathamboo et al. [63], Olsen et al. [64], Rukasha et al. [65], Simblett et al. [66], Johansson et al. [67], and Ryvlin et al. [68] evaluated the challenges faced by wearable sensors for the care of patients with epilepsy. Finally, Naganur et al. [69], Ong et al. [70], Cullen et al. [71], Nielsen et al. [72], Beniczky et al. [73], Shum et al. [74], Ó Breasail et al. [75], de la Fuente García et al. [76], Machado et al. [77], Gillani et al. [78], Anderson et al. [79], Bruno et al. [80], Vinals et al. [81], Kurada et al. [82], Klimova et al. [83], and Ienca et al. [84] comparatively evaluated a series of wearable devices that use AI to monitor motor impairments caused by AD. The purpose of the review was to identify gaps and research opportunities to better care for patients with AD.

The four underlying differences of our research, if compared to the literature reviews previously mentioned, can be summarized as follows: First, our review focuses on currently available commercial and non-commercial wearable devices for monitoring NDD-related motor impairments. Second, we classify these devices according to their characteristics. Third, we identify both NDD-related motor impairments and the sensors used for monitoring these impairments. Finally, we discuss the Food and Drug Administration (FDA) status of each of the reviewed wearable technologies. The focus of this paper is NDD-related motor impairments. In this sense, our four main objectives are (1) to identify currently available commercial and non-commercial wearables for monitoring NDD-related motor impairments, (2) to list the characteristics of such devices, (3) to rate the top most used devices for monitoring motor impairment in patients with NDDs, and (4) to determine the FDA status of such devices.

## 3. Methods

This review examines the quantitative and qualitative aspects of research on wearable devices (Commercial and Non-commercial) for monitoring the motor affectations caused by neurodegenerative diseases. We follow the three stages of the methodology proposed by Brereton et al. [85]. It is considered a reference method to carry out systematic reviews of the literature. The first stage of this methodology is planning, in which the need to carry out a review of the literature is determined, research questions are formulated, and the protocol to be followed is reviewed. In the second stage, the search strategy is determined, the primary studies are selected, and the data of interest are extracted and synthesized. In the final stage, the results are obtained, their validity is verified, and conclusions are made.

### 3.1. Research Questions and Motivations

Four research questions were posed to achieve the objectives of this review. Table 1 shows the five research questions.

### 3.2. Search Strategy

The following five digital libraries were used to search for the primary studies: JMIR, Springer Link, Science Direct, Annuals Reviews, and Wiley Online. Google Scholar search engine was used to expand the results obtained. The libraries were selected based on their prestige and popularity with the research community, they all provide access to a wealth of digital literature, and they are peer-reviewed studies. The search was conducted based on keywords. The search period used was from 2012 to mid-2022 (The last 10 years). Table 2 shows the keywords used in this review.

**Figure 3:**
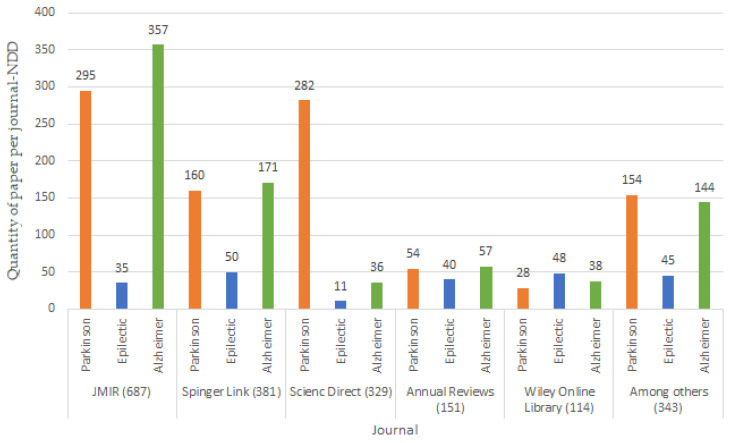
Research papers by digital libraries.

From a combination of the keywords shown in Table 2, search strings were formed using the “AND” and “OR” connectors as follows: ((wearables devices) OR (Sensors)) AND ((Neurodegenerative diseases) OR (mental diseases)) between the years 2012 and 2022. Figure 3 graphically shows the relevant search results beginning in 2005, and the top five results were: 687 JMIR, 381 Springer Link, 329 Science Direct, 151 Annuals Reviews, 114 Wiley Online, 343 among others, such as BioMed Central, Google Scholar, IOP Science, Hindawi, IEEE Xplore, AHA Journals, MDPI, Clinicals Trials and JACC.

### 3.3. Selection of Primary Studies

This research is a review of the wearable devices used for monitoring NDD-related motor impairments. The review follows the PRISMA guidelines [86] to ensure the correct organization and clarity of our findings.

#### 3.3.1. Collection of Sources

We initially obtained our search results from 2005 sources of interest across multiple databases. After refining the search to comprise research from 2010 and onwards, we discarded 236 results and left 1769 potential sources for review.

#### 3.3.2. Inclusion and Exclusion Criteria

*Inclusion criteria*: The literature related to the topic of monitoring NDD-related motor impairments were published from 2010 to 2022, and the particular topics related to (1) wearables sensor devices and (2) commercial and non-commercial wearables were considered. *Exclusion criteria:* The literature that was discarded was for (1) manuscripts written in languages different than English, (2) letters and reports, (3) conference and symposium proceedings, (4) non-primary studies, and (5) non-peer-reviewed sources.

#### 3.3.3. Information Sources

Table 3 shows the sources of information where the search was carried out based on the related term (Category).

#### 3.3.4. Search Strategy

We combined the research keywords with Boolean connectors (AND/OR) to limit the number of results obtained in the search. The search keywords were extracted from the research questions. Below we list the intermediate search phrases used to find the terms that were used in subsequent queries:

1. Main motor impairments related to NDDs.
2. Wearable devices used to monitor NDD-related impairments.
3. Commercial and non-commercial wearable devices for monitoring NDDs.
4. Commercial sensors used in wearable devices.
5. FDA status of commercial wearable devices.

From the list of queries above, new terms were found that were relevant to this study.

#### 3.3.5. Selection Process

**Figure 4:**
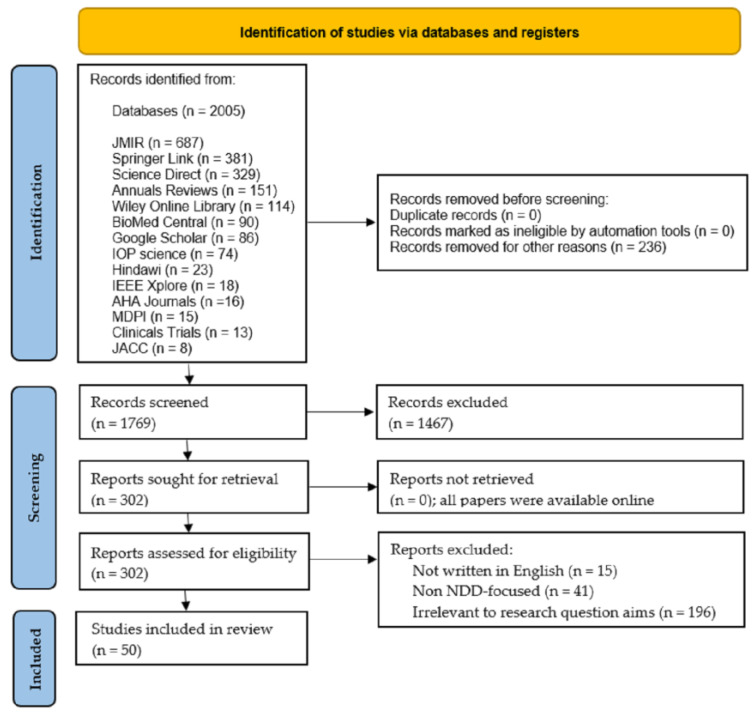
PRISMA flow diagram for the literature search.

Four experts in the field (SMEs) participated in this selection process. Figure 4 shows the flowchart of our PRISMA-based methodology, which shows the process of how the 50 articles were obtained that meet the inclusion criteria set forth above. The 50 selected articles were downloaded in their entirety from the following databases: MDPI (11), Science Direct (11), Google Scholar (8), PubMed (8), IEEE Xplore (5), Biomed central (2), Springer Link (2), Wiley Online Library (2), and ACM (1).

#### 3.3.6. Data Collection and Analysis

From the 50 selected articles, relevant information for this study was collected and organized in structured tables. Four SMEs supervised the analysis to extract relevant and interesting information, such as motor impairment, brand and model of wearable device, operational features, used sensor(s), and FDA status.

### 3.4. Data Extraction

We extracted two types of data in each of the selected studies: (1) bibliographic; for example, title, authors, objectives, and repository and (2) content, with which the research questions raised in this review were answered. The following section presents the results obtained from the studies considered.

## 4. Results

This review analyzes commercial and non-commercial wearable devices used for monitoring NDD-related motor impairments. The commercial wearables considered in this review comprise both pre-sale and commercially available devices. Prototype and investigational wearable devices were categorized as non-commercial. In total, we found 22 commercial and 28 non-commercial wearable devices for monitoring NDD-related motor impairments.

### 4.1. RQ1. Which Are Commercial Wearables for Monitoring NDD-Related Motor Impairments Currently Available?

Table 4 summarizes our findings in this review regarding commercially available wearable devices. Practically the majority of the wearable devices found are aimed at monitoring the motor deficiencies caused by NDDs during the daily activities of the patient; however, not all of these devices have been approved by regulatory agencies, such as the US Food and Drug Administration (FDA), which regulates the sale of such wearable devices and provides guarantees to consumers that they are safe and effective in their intended use.

### 4.2. RQ2. Which Are the Top Most Used Commercial Wearables for Monitoring Motor Impairment in Patients with NDDs?

**Figure 5:**
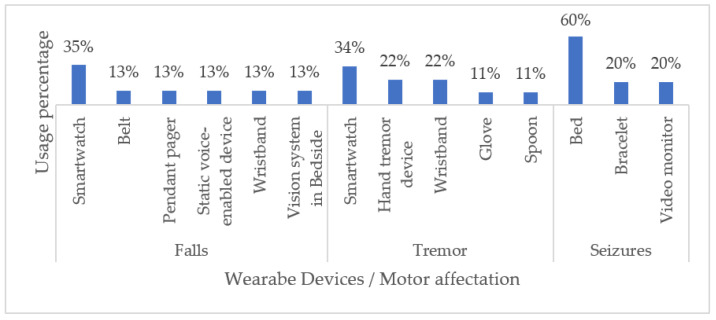
Classification of Commercial Wearable Devices by NDD-Related Motor Disabilities.

Table 4 summarizes our findings regarding the most important characteristics of each device reviewed, including its FDA status. Most of the commercially available wearable devices used for monitoring NDD-related motor impairments are smartwatches and beds (60%). Even though smartwatches have been best implemented to monitor tremors and seizures (35% and 34%, respectively), the work continues to improve the performance of other wearable devices. In this sense, fabrics and tattoos have also emerged as non-invasive alternatives for monitoring patients during daily activities. Figure 5 illustrates our classification of wearable devices with respect to the NDD-related motor impairments that they commonly monitor.

**Figure 6:**
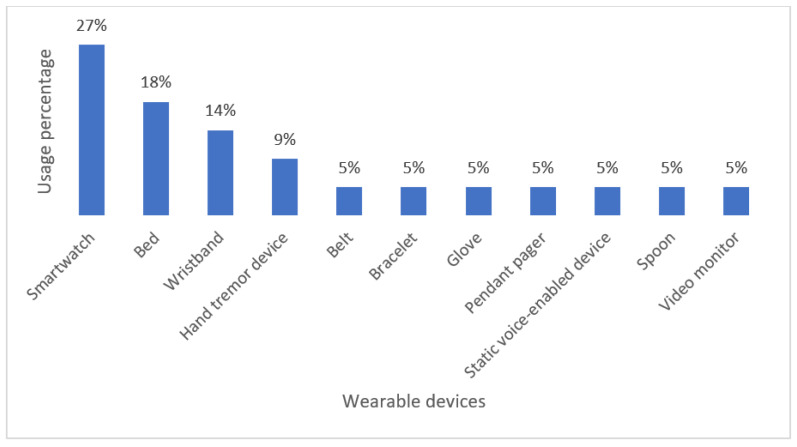
Classification of Commercial Wearable Devices for Monitoring NDD-Related Motor Impairments.

As Figure 6 illustrates, the smartwatch is the most used to monitor NDD-related motor disabilities. Current scientific and technological progress has made it possible for companies to manufacture increasingly compact wearable devices that can be easily worn on the body while promising favorable and convenient results in terms of health monitoring. However, much better performance in monitoring and patient care remains a challenge for such devices.

### 4.3. RQ3. Which Are Technical Characteristics of Non-Commercial Wearables for Monitoring NDD-Related Motor Impairments Currently Available?

In this review, non-commercial wearable devices comprised prototype devices and devices developed solely for research purposes. Table 5 summarizes our findings in terms of the following aspects:

- Year of publication of the research;
- Aimed NDD;
- Type of wearable device;
- Brief research description;
- Sensors or technology used;
- Real-time device monitoring capability.

**Figure 7:**
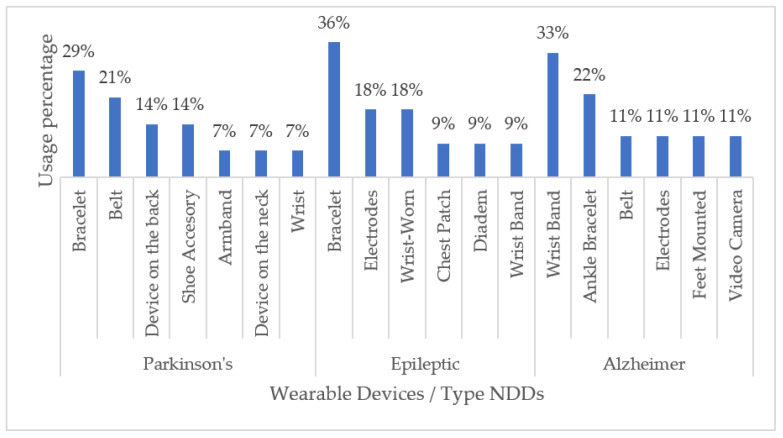
Non-Commercial Wearable Devices for Monitoring NDD-Related Impairments.

With respect to commercial handheld devices, non-commercial ones are commonly developed for a particular NDD. Overall, we found that devices to be worn on the wrist, such as bracelets, are the most used for monitoring motor disabilities related to Parkinson’s disease (29%), Alzheimer’s disease (33%), and epilepsy (36%). Generally, wrist wearable devices are chosen as the first option with respect to other options of wearable devices due to a good relationship between the comfort-accuracy of said device. Figure 7 shows a graphical summary of our findings.

Real-time monitoring of patients with conditions caused by NDD allows health professionals and patients themselves to obtain timely information on their health status during daily activities. Table 6 summarizes the findings of this review with regard to non-commercial wearables.

Table 7 summarizes our findings regarding the technologies implemented in non-commercial wearables to monitor NDD-related disabilities. As can be seen, the technological implementation based on accelerometers and AI (through ML techniques) is the most used. Accelerometers are implemented in bracelets or wristbands, thus implying once more that the comfort–accuracy ratio of such devices contributes to their popularity.

### 4.4. RQ4. Which Are the FDA Status of Commercial Wearables for Monitoring NDD-Related Motor Impairments Currently Available?

**Figure 8:**
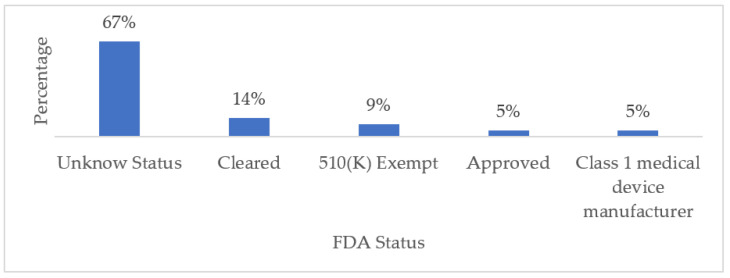
FDA Status of Commercial Wearables Used for Monitoring NDD-Related Motor Impairments.

We considered five statuses or categories to classify the reviewed commercial wearable devices: (1) Approved, which means that the benefits of the wearable device outweigh the known risks for the intended use. (2) Class I, the device is not intended for use in supporting or sustaining life or to be of substantial importance in preventing impairment to human health but may not present a potentially unreasonable risk of illness or injury. (3) 510 (K) Exempt means that the FDA is requesting notification, along with evidence, that the medical device intended to be marketed is safe and effective prior to a company commercializing its product. (4) Cleared, which means that the manufacturer can demonstrate that their product is substantially equivalent to another (similar) legally marketed device that already has FDA clearance or approval; and finally, (5) Unknown, which means that we did not find any information on the FDA status of a particular device. In this sense, it is worth mentioning that some manufacturers do not publicly disclose FDA-related information about their products. Our findings revealed that 32% of the wearable devices have some degree of FDA approval, while for the remaining 68%, we did not find any related information. Figure 8 and Table 8 summarize these results.

### 4.5. RQ5. What Are the Gaps in the Monitoring of NDDs Using Commercial Wearables Devices? And How Are These Gaps Covered by the Non-Commercial Wearables Devices?

Based on the information presented in Table 3, it can be concluded that the gap that commercial wearable devices have to cover can be summarized in two ways: (1) Improving the accuracy of the information collected from the user in any environment in which the user; that is to say, that the information that is collected from the user must be in an environment of the normal activity of the latter; that is, when the user is sleeping, walking, running, in the office, among others since from the aforementioned data it will be possible to obtain relevant results of the user’s health and (2) Build commercial wearables devices that are imperceptible to the user. The reason that most of the devices in the form of watches and bracelets are found on the market is because that technology offers a high relationship between comfort–accuracy of the data obtained from the patient compared to traditional visits to a health center. Connecting to a device to passively monitor any sign of the patient’s health does not guarantee relevant results. The Research Centers are public, private, or mixed organizations dedicated to the generation of knowledge to solve the needs of society in general, including health. Currently, there are research centers where non-commercial wearables devices are developed based on technology in the form of tattoos, smart wearables, and biosensors, among others, with which the gap that currently exists in commercial wearables devices is expected to decrease.

This review provides valuable information on wearable devices for monitoring motor impairments suffered by patients with NDDs. First, our findings revealed that commercial wearable technologies generally focus on measuring falls, tremors, and seizures via smartwatches. As for non-commercial wearables, bracelets and wristbands are the most popular and usually rely on an accelerometer for monitoring. Both commercial and non-commercial wearable devices are significantly more used on the patient’s wrist because wrist-worn devices offer a significantly good comfort-accuracy ratio. The ideal wearable device is one that makes a precise measurement of the monitoring performed and that, in turn, is transparent to the user and/or patient. That is, it is a wearable device that does not interfere with the patient’s day-to-day activities. In this sense, tattoos, rings, glasses, and clothing (among others) have emerged as wearable trends that are almost completely imperceptible to the patient. Moreover, thanks to the implementation of AI in the development of these devices, technology based on video monitoring promises to be encouraging in optimizing the monitoring of NDD-related motor impairments since it will not be necessary to wear any device to monitor our health.

## 5. Discussion

### 5.1. Challenges and Trends

Current commercial wearable devices are aimed at monitoring factors such as physical activity, heart rate, caloric intake, diet, reading texts, answering calls, and sending notifications. However, as their main critical aspect, NDDs are difficult to diagnose at their early stages, which implies that, despite their great usefulness in the monitoring and follow-up of some chronic-degenerative diseases, current wearable devices are not specifically intended to support NDD care and/or treatment. In this sense, the following challenges must be addressed in the monitoring of NDDs and NDD-related impairments:

1. Research new risk factors (biomarkers and/or biometric factors) for NDDs.
2. Optimize monitoring and measuring algorithms.
3. Develop non-invasive and transparent technology for users.
4. Optimize the connectivity of the data transmitted/received by these devices through wireless networks and personal area networks (PANs).
5. Develop devices that optimally manage power consumption and rely on alternative sources of energy, such as solar energy.
6. Guarantee the security and privacy of patient data.

In recent years, the medical and scientific communities have thrived in identifying biomarkers (i.e., biologically measurable parameters) that help timely and accurately indicate the development of an NDD. Ideally, a biomarker should be easily accessible, highly sensitive and specific, and must correlate with high levels of NDD progression. Advances in biomarker research have given rise to wearable devices that can form part of the body. An example of this is implantable, wearable technology, which consists of devices such as adhesive patches or electronic chips that are implanted in the human body through surgery so that they become part of the user permanently.

Trends in NDDs research already provide solutions in the form of wearable devices that collect biometric data by recording data on patient behavior and habits (e.g., when typing, scrolling or moving fingers) to timely identify and treat neuromotor or neurodegenerative diseases, such as PD or AD. Additionally, current technologies for monitoring NDD-related impairments (motor and cognitive) include Augmented Reality (AR), audio systems, e-skin devices, e-textiles, ingestible and insertable technologies, neural interfaces, and chemical biosensors. AR-based wearable devices can provide additional information that cannot normally be seen with the naked eye. Other areas of AR include tourism, art exhibitions, and manufacturing. Regarding audio systems, conventional (wired and wireless) speakers-headphones and high-quality equalizers can be integrated as part of the AR system to improve patient immersion. As for e-Skin or nanopatches, their technological development is similar to “artificial skin”, with the same mechanical properties as human skin. This attribute significantly increases the detection area of wearable devices that use this type of sensor. The use of this technology is not limited to humans, as it also extends to robotic systems, with the intention of providing them with near-human perception abilities to be able to use them, for example, during surgical interventions.

Unlike the e-Skin concept, e-Textiles or smart fabrics cover a significant part of the body. In this case, sensors, circuits, or input/output devices are integrated into the fabric that is part of the patient’s daily-use garments. As for ingestible and insertable technologies, they are inserted into the human body or administered as medicine in a capsule form. They comprise sensors, microprocessors, and controllers, among others, that collect, process, and send biomedical signals of patient health data. Ingestible and insertable wearable technology is considered the future in the monitoring and diagnosis of diseases. Neural interfaces aim at supporting the care of patients with complex medical conditions via an interpretation of brain function to diagnose and monitor the disease.

Chemical biosensors such as saliva, tears, and sweat are biological fluids with multiple physiologically relevant chemical components. Saliva is a complex biofluid with numerous components that permeate blood through transcellular or paracellular pathways. Saliva analysis is a non-invasive option to analyze blood and monitor the emotional, hormonal, nutritional, and metabolic state of the human body. On the other hand, the concentration levels of glucose, lactate, or neurotransmitters in tears are of vital importance for health monitoring. Finally, sweat is commonly used for both monitoring fluid and electrolyte loss and managing certain diseases. In this sense, it is possible to monitor non-invasively and in real-time relevant aspects in patients with NDDs. Finally, the reality of wearables in 2022 is marked by the predominance of smart bracelets and smartwatches. As time goes by, these devices are likely to pave the way for other wearable technologies (e.g., smart glasses, smart chips and cybernetic implants) with greater monitoring capabilities.

### 5.2. Emerging Solutions

The current technological revolution is driven by digitization and, above all, the development and application of AI in multiple fields. AI technologies can learn and act in response to the environment, which simulates a “mental” process that allows machines to make decisions and perform tasks that are originally human or that people are not even capable of doing on their own. AI has great potential to improve NDD detection and monitoring thanks to intelligent algorithms that are capable of recognizing and analyzing unstructured data to convert them into relevant and useful information. Additionally, as a branch of AI, ML enables pattern recognition and/or the ability to continuously learn and make predictions based on data to make adjustments automatically.

The implementation of AI altogether with ML is present in different areas of the scientific panorama, such as medicine. Hence, the synergy between ML and AI has allowed for faster and more accurate diagnosis of existing diseases, including cancer, dementia, and Alzheimer’s disease, which is a disease that modifies the brain and causes alterations that affect memory, understanding, judgment, behavior, and functional activity.

AD is one of the greatest health challenges that health systems face around the world, both because of the impact it has on patients and their families and because of the high resources needed to treat it. Addressing NDDs with ML and AI algorithms has made it possible to identify the first markers of AD with more than 99% accuracy. In fact, by evaluating the brain scans of older adults, algorithms can detect subtle changes that often occur before diagnosis, allowing doctors to offer early treatment to high-risk individuals.

### 5.3. Limitations

This review has four main limitations: (1) We did not consider scenarios or comparative clinical studies that assess the quality of life of patients when using multiple portable devices, (2) mobile applications associated with the reviewed devices were not analyzed, (3) studies on consumer acceptance of wearable devices were not reviewed, and (4) the medical-grade accuracy and reliability of the reviewed wearable devices could not be established due to limited information on their FDA status.

## 6. Conclusions

The wearable devices that are used for the remote monitoring of NDD-related motor impairments include built-in sensors and can be used during daily activities. Our findings revealed that falls are most monitored for PD, whereas tremor monitoring is predominant in wearable devices for epilepsy and AD management. Only wearable devices with suitable, well-calibrated, and coordinated sensors can provide relevant medical monitoring data. The use of a certain type of wearable device over another largely depends on the condition to be monitored; however, in general, the most frequently cited wearable devices in the literature include smartwatches and wristbands. Additionally, this review discusses the medical-grade accuracy and reliability of the cited wearable devices by listing their FDA status. In this regard, we found that 67% of the cited devices held an unknown FDA status, 28% were partially approved, and only 5% were fully FDA authorized.

Overall, NDDs cause two types of impairments: cognitive and motor. The scope of this research is limited to wearable devices monitoring only NDD-related motor impairments. In this sense, our main findings can be summarized as follows: commercial wearable devices mainly monitor falls, tremors, and seizures. Additionally, 35% of these devices are wrist-worn devices in the form of either a smartwatch or a band. Finally, only 33% of the reviewed commercial wearables hold an FDA approval status (approved, partially approved, authorized). Regarding non-commercial wearable devices, wrist-worn are also ranked as the most common due to their good comfort–accuracy ratio. Finally, thanks to the implementation of AI in mobile device software development, monitoring technologies based on video monitoring promise to be encouraging in terms of optimizing the monitoring capabilities of current wearable devices, thus implying that in the future, patients may not need to wear any type of device to still be able to monitor aspects of their health.

## Figures and Tables

**Table 1:** Research Questions.

Research Question (RQ)	Question	Motivation
1	Which are commercial wearables for monitoring NDD-related motor impairments currently available?	To identify the main commercial wearables for monitoring NDD-related motor impairments currently available.
2	Which are the top most used commercial wearables for monitoring motor impairment in patients with NDDs?	To identify the top most used commercial wearables for monitoring motor impairment in patients with NDDs.
3	Which are the technical characteristics of non-commercial wearables for monitoring NDD-related motor impairments currently available?	To identify the technical characteristics of non-commercial wearables for monitoring NDD-related motor impairments currently available.
4	Which are the FDA status of commercial wearables for monitoring NDD-related motor impairments currently available?	To determine if the patient can confidently use the commercial wearables for monitoring NDD-related motor impairments currently available.
5	What are the gaps in the monitoring of NDDs using commercial wearables devices? How are these gaps covered by non-commercial wearables devices?	To identify the areas of opportunity that must be strengthened in commercial wearables devices.

**Table 2:** Keywords and related concepts used in this literature review.

Area	Keywords	Related Concepts
Neurodegenerative Disease	Motor impairments	Falls
Mental health	Wearables and sensors devices	Tremor
		Seizures
		Parkinson
		Epileptic
		Alzheimer

**Table 3:** Table 3 shows the sources of information used.

Category	
Information Technologies	Healthcare
Google Scholar, Hindawi, IEEE Xplore, IOP science, JACC, MDPI, Nature, Science Direct, Springer Link, and Wiley Online Library.	AHA Journals, Annual Reviews, BioMed Central, Clinical Trials, JMIR, and PubMed

**Table 4:** Commercial Wearable Devices for Monitoring NDD-Related Motor Impairments.

Motor Disability	Device Type	Device Brand	Device Model	Monitoring Features	Sensors Used	FDA Status/Year/AP (AP: Accuracy Percentage)	Android Compatibility
Falls	Smartwatch	Apple	Watch Series 7 [87]	Detect user idleness for about a minute. It begins a 30-s countdown while tapping you on the wrist and sounding an alert.	Blood oxygen, electric HR, optical HR, GPS, compass, microphone, altimeter, and horn.	Approved/2018 /98%/(Only for ECG)	No
	Smartwatch	Watchseniors	Plus 4 G [88]	Detect falls, trajectories, and user location.	Blood pressure sensor, temperature sensor, and heart rate sensor	Unknown	Yes
	Smart Phone	Freeus	FallSafety [89]	Detect user falls with automatic generation of alerts to the emergency systems.	Information not available	Unknown	Yes
	Belt	Fallskip	TF11-MP005-ES [90]	Assess the risk of falling in older adults.	IMU (Inertial Measurement Unit)	Unknown/Unknow/75%	No
	Pendant pager	Neki	Nock Senior [91]	Fall detector with GPS locator monitorable with its application on the cell phone.	Fall detector sensor	Unknown	Yes
	Static voice-enabled device	Amazon	Alexa together [92]	Virtual assistant equipped with functions that help the user in case of falls.	Speech processor	Unknown	Yes
	Wristband	MyNotifi^®^	MYNOTIFI FALL DETECTION SYSTEM [93]	Detect falls and alert the user’s family and friends if a fall occurs.	AI sensor	Class I (Exempt)/2020/73%	Yes
	Vision system in Bedside	VIRTUSENSE^TM^	VSTOne [94]	Predict a crash around 30–65 s before it occurs.	AI and LiDAR sensors	Unknown/Unknown/98%	No
Tremor	Smartwatch	Microsoft	Emma Watch [95]	Reduce tremors associated with Parkinson’s disease.	Movement is regulated by a sensorimotor feedback loop involving the perception of movement and position of the body.	Unknown	No
	Smartwatch	Detekt	PKG watch (The Parkinson’s KinetiGraph) [96]	Analyze user movements throughout the day and output a graph that allows the doctor to analyze and compare user movement speed and overall user capability to move throughout the day.	Gyroscopic stabilization	510(k) Cleared/2014/96%	No
	Smartwatch	Parkinson Smartwatch	Parkinson Smartwatch [97]	User (i.e., patient) manually records information on their perception of well-being during the day (for example, after taking their medication). The information that is recorded on the device is sent to the cloud, where it is stored. The user and their doctor have online access to the graphs of the data recorded from anywhere in the world using a computer, tablet, or mobile phone.	Information not available	Unknown	No
	Glove	Steadiwear	Steadi-Two [98]	Reduce tremor magnitude using two magnets to control a disk that moves in the opposite direction of the tremor.	The technology is based on a seismic design and works similarly to a see-saw in a park. The disk, which is controlled by magnets, responds to the tremor by providing an equal and opposite force, lowering its magnitude.	Class I/2021/80%	No
	Handheld Therapeutic device	VILIM	VILIM ball [99]	Reduces the hand tremors of the patient while performing daily tasks.	Embedded algorithm that analyzes tremors and adapts to each patient’s symptoms individually.	Unknown	Yes
	Hand Tremor Device	Five Microns^®^	Tremelo [100]	Reduces intermediate-degree tremors in the arms and hands by 85 to 90%.	Non-invasive and mechanical (no electricity: no batteries) device relying on vibration absorbers and tuned mass damper.	Class 1/Unknown/90%	No
	Spoon	GYENNO	GYENNO SPOON [101]	Stabilize unwanted tremors by 85% to stop intentional hand movement and help people with PD or tremors to eat more easily.	Intelligent rehabilitation robotics, Intelligent high-speed servo control system, algorithm technology of unmanned aerial vehicles, and self-adapted ML.	Unknown	Yes
	Wristband	Parkinson’s KinetiGraph (PKG)	PKG^TM^ [102]	Collect data on motor disabilities and other complications caused by PD (e.g., slowness of movement, tremor, stiffness).	Sensors that monitor the wearer’s activity and buzzes for medication reminders.	Cleared/Unknown/Unknown	No
	Wristband	The Cala Trio therapy	Cala Trio™ [103]	Deliver electrical stimulation—also known as neuromodulation—to the nerves in the effective wrist. The stimulation disrupts the tremor network in the brain and delivers meaningful tremor reduction in the affected hand.	Information not available	Cleared Class II /2020/68%	No
Seizures	Bed	EpiUSA	Emfit Movement Monitor [104]	Nightly monitoring of a person’s movements to alert their caregivers if necessary.	Motion sensor installed in a pad, which is installed under the patient’s mattress.	Unknown	No
	Bed	MedPage	BMA-01 [105]	Detect certain types of movements (e.g., muscular spasms) that people make while sleeping using a movement-sensing alarm.	Sophisticated software algorithms that continually analyze the signals produced by a special sensor positioned under the bed mattress, even a memory foam type.	Unknown	No
	Bed	Epi USA	Emfit [106]	Detect most movements, including light movements in patients with epileptic disease. The company states that this product is also suitable for small children.	Flexible and durable bed sensor, a bedside monitor, a bed clip, and a wall bracket. The movement monitor detects movement over a preset amount of time and triggers an alarm if a person moves more than it expects.	Unknown	No
	Bracelet	Empatica	Embrace 2 [107]	Detect seizures	Information not available	FDA cleared/2018/98%	Yes
	Video monitor	SAMi	SAMi-3 [108]	Process and record patient movements in real-time. Send alerts to caregivers in case of patient seizures.	Information not available	Unknown	Yes

**Table 5:** Non-Commercial / Research Wearables for Monitoring NDD-Related Motor Impairments.

NDD Type	Device Type	Research Description	Sensors or Technology Used	Real-Time Monitoring
Parkinson	Bracelet (2017)	Use wearable sensors to quantify doses in patients with PD to address motor affections such as tremors, bradykinesia, and dyskinesia [109].	Wrist and ankle motion sensors	Yes
Parkinson	Bracelet (2018)	Evaluate a fall prediction test using body sensors in patients with PD [110].	Inertial sensor and software system (Kinesis QTUG™, Kinesis Health Technologies, Dublin, Ireland)	Yes
Parkinson	Bracelet and Belt (2021)	Evaluate motor disabilities in patients with PD [111].	Accelerometer	Yes
Parkinson	Shoe accessory (2020)	Using a 3D accelerometer, they validated a pair of pressure insoles in shoes to detect walking problems in patients with PD [112].	Accelerometer 3D and pressure insoles	Yes
Parkinson	Armband	Propose a wearable device for the diagnosis of motor affections such as rigidity, tremor, and bradykinesia in patients with PD [113].	Sensor system composed of a force sensor, three inertial measurement units (IMUs), and four custom mechanomyography (MMG) sensors	Yes
Parkinson	Bracelet and Belt (2021)	Evaluate the data obtained from a group of patients with PD by means of wearable sensors to quantify the severity of symptoms in the extremities of the patients [114].	Accelerometer	Yes
Parkinson	Wrist (2020)	Validate a mechatronic wearable device that seeks to mitigate wrist stiffness in patients affected by PD [115].	One actuated joint and four passive revolute joints with a high overall intrinsic back drivability.	Yes
Parkinson	Shoe accessory and Belt (2022)	Monitor and evaluate gait in patients with PD through a portable physiograph [116].	Pressure sensors, electromyography (EMG) sensors, and accelerometers.	Yes
Parkinson	Device on the back (2022)	Evaluate the performance of a device to monitor and improve postural alignment, balance, and gait in patients with PD [117].	Device Up Right Go	Yes
Parkinson	Device on the neck and back (2019)	Propose a method to estimate stooped posture through sensors (i.e., accelerometers) mounted on the patient’s neck or upper back [118].	Accelerometer	Yes
Epileptic	Bracelet (2021)	Evaluate the performance of the bracelet in detecting seizures through algorithms implemented with ML using multisignal biosensors worn on the patient’s wrist and ankle [119].	Wrist- and ankle-worn multisignal biosensors in conjunction with machine learning algorithms (MLAs)	No
Epileptic	Chest patch (2019)	Evaluate seizure detection through heart rate variability using a portable electrocardiography device [120].	Portable Electrocardiogram (ECG) in conjunction with algorithms implemented in LabView	No
Epileptic	Wristband (2019)	Evaluate a portable system based on accelerometry to detect tonic–clonic seizures [121].	Inertial sensors	No
Epileptic	Bracelet (2022)	Propose an automated method based on machine learning to classify seizures [122].	Accelerometer and gyroscope	Yes
Epileptic	Bracelet (2022)	Develop a system to detect seizures (epileptic / non-epileptic) using wearable sensors [123].	Electroencephalography (EEG), Electromyography (EMG), and ECG	Yes
Epileptic	Electrodes (2022)	Monitor patients with epilepsy disease to propose effective strategies for seizure detection [124].	EEG, ECG, and accelerometer	Yes
Epileptic	Wrist-Worn (2018)	Develop a wireless monitoring system (with an accelerometer as a sensor) worn on the patient’s wrist for seizure detection [125].	Accelerometer	Yes
Epileptic	Wrist and ankle Bracelet (2022)	Investigate the effects of anticonvulsant medications monitored by a wearable device in patients with epilepsy [126].	Body temperature sensor, optical, infrared sensors, and a 3D accelerometer and gyroscope.	Yes
Epileptic	Diadem (2022)	Evaluate the accuracy of absence seizure detection using an electroencephalographic wearable device [127].	EEG	No
Epileptic	Wrist-Worn and Electrodes (2017)	Develop a wearable system that detects seizures and alerts patient caregivers [128].	EEG, gyroscope, 3D accelerometer, optical, infrared sensors, and body temperature.	No
Alzheimer	Ankle Bracelet (2018)	Evaluate an algorithm to monitor and record gait movements in patients with AD [129].	Accelerometer and gyroscope	No
Alzheimer	Electrodes (2022)	Develop and evaluate a multiclass classification system for AD based on a commercial EEG acquisition system that uses sixteen channels [130].	EEG	No
Alzheimer	Wrist and ankle Bracelet (2019)	Review wearable devices that monitor and control posture and gait in patients with dementia [131].	Accelerometer and gyroscope	No
Alzheimer	Video camera (2018)	Develop a platform to support patients suffering from impaired facial perception with an assistive intelligence device [132].	Algorithm- Facial Perception Model	No
Alzheimer	Wrist band (2015)	They developed a localization band targeted at people suffering from memory diseases [133].	GPS and global system for mobile (GSM) communication	Yes
Alzheimer	Wrist band (2018)	Determine whether characteristics extracted from arterial pulse waves (PWs) measured by wearable sensors could be useful for stratifying patients at risk of AD [134].	Photoplethysmography (PPG)	No
Alzheimer	Feet mounted (2014)	Develop gait and balance analysis algorithms for the diagnosis of patients with AD [135].	Inertial sensor	No
Alzheimer	Belt (2016)	Investigate ML classifiers applied in postural control in patients with AD [136].	Multiple Layer Perceptrons (MLPs), accelerometer, and gyroscope	No

**Table 6:** Non-Commercial Wearables with Real-Time Monitoring Capabilities.

NDD Type	Real-Time Monitoring	Distribution
Alzheimer’s disease	Yes	13%
	No	87%
Epilepsy	Yes	50%
	No	50%
Parkinson’s disease	Yes	100%
	No	0%

**Table 7:** Technology in Non-Commercial Wearables for Motoring NDD-Related Motor Impairments.

NDD Type	Sensor/Technology Used	Usage Percentage
Parkinson’s disease	Accelerometer	30%
	Mechanical joint	14%
	Pressure sensors	14%
	MMG sensors	7%
	EMG	7%
	Force sensor	7%
	Inertial sensor	7%
	Software system	7%
	Wrist and ankle motion	7%
Epilepsy	Accelerometer	21%
	EEG	21%
	Body temperature sensor	11%
	ECG	11%
	Gyroscope	11%
	Biosensors with Machine Learning Algorithms (MLAs)	5%
	EMG	5%
	Inertial sensors	5%
	Infrared sensors	5%
	Optical sensors	5%
Alzheimer’s disease	Accelerometer	20%
	Gyroscope	20%
	MLAs	20%
	EEG	10%
	GPS and GSM	10%
	Inertial sensor	10%
	PPG	10%

**Table 8:** NDD-Related Motor Impairments Monitored by Commercial Wearable Devices.

Motor Disabilities	FDA Devices	Non-FDA Devices
Falls	38%	62%
Tremor	33%	67%
Seizures	20%	80%

## Data Availability

Not applicable.
